# The final frontier: ecological and evolutionary dynamics of a global parasite invasion

**DOI:** 10.1098/rsbl.2022.0589

**Published:** 2023-05-24

**Authors:** Nadine C. Chapman, Théotime Colin, James Cook, Carmen R. B. da Silva, Ros Gloag, Katja Hogendoorn, Scarlett R. Howard, Emily J. Remnant, John M. K. Roberts, Simon M. Tierney, Rachele S. Wilson, Alexander S. Mikheyev

**Affiliations:** ^1^ School of Life and Environmental Sciences, Behaviour, Ecology and Evolution Lab, The University of Sydney, NSW 2006, Australia; ^2^ School of Life and Environmental Sciences, The University of Sydney, NSW 2006, Australia; ^3^ School of Natural Sciences, Macquarie University, Macquarie Park, NSW 2109, Australia; ^4^ Hawkesbury Institute for the Environment, Western Sydney University, NSW 2753, Australia; ^5^ School of Biological Sciences, Faculty of Science, Monash University, Clayton Victoria 3800, Australia; ^6^ School of Agriculture, The University of Adelaide, Food and Wine, Adelaide SA 5005, Australia; ^7^ Commonwealth Scientific & Industrial Research Organisation, Canberra 2601, ACT, Australia; ^8^ Hawkesbury Institute for the Environment, Western Sydney University, Richmond, NSW 2753, USA; ^9^ School of Biological Sciences, University of Queensland, St Lucia, QLD 4072, Australia; ^10^ Research School of Biology, Australian National University, Canberra, ACT 26000, Australia

**Keywords:** pollination, invasive species, mites, *Apis*, viruses

## Abstract

Studying rapid biological changes accompanying the introduction of alien organisms into native ecosystems can provide insights into fundamental ecological and evolutionary theory. While powerful, this quasi-experimental approach is difficult to implement because the timing of invasions and their consequences are hard to predict, meaning that baseline pre-invasion data are often missing. Exceptionally, the eventual arrival of *Varroa destructor* (hereafter Varroa) in Australia has been predicted for decades. Varroa is a major driver of honeybee declines worldwide, particularly as vectors of diverse RNA viruses. The detection of Varroa in 2022 at over a hundred sites poses a risk of further spread across the continent. At the same time, careful study of Varroa's spread, if it does become established, can provide a wealth of information that can fill knowledge gaps about its effects worldwide. This includes how Varroa affects honeybee populations and pollination. Even more generally, Varroa invasion can serve as a model for evolution, virology and ecological interactions between the parasite, the host and other organisms.

## 1. Introduction

The impacts of large-scale biological invasions are hard to assess because invasions are difficult to predict, and pre-invasion baseline data are often not collected before it is too late.

However, biological invasions are opportunities to observe evolutionary changes and their ecological consequences in real time. They can inform theory about how organisms respond to novel ecological conditions [1,2].

Exceptionally, in 2022, *Varroa destructor* mites (hereafter Varroa), ectoparasites of honeybees that are responsible for worldwide colony losses, were detected in Australia, which has remained the last continent to be colonized by this pest. Originally found in Asia on the eastern honeybee (*Apis cerana*), Varroa switched to western honeybees (*A. mellifera*) in the mid-twentieth century and spread to Africa, Europe and the Americas [3,4]. In Europe and North America, they caused large-scale population declines, particularly among unmanaged honeybees, in large part by spreading deadly viruses [3]. Despite decades of study, major gaps in our understanding of how Varroa affects bees and the ecosystem at large remain [5]. While aggressive efforts to contain Varroa's spread in Australia are underway, the risk of establishment from this or a future incursion is high.

While a number of studies have been able to conduct pre- and post-Varroa comparisons, or even trace changes along the invasion front (e.g. [6–8], among many others), they all have limitations in terms of sampling strategy, the technology available at the time, or focus on particular elements of the bee–Varroa–virus interaction at the expense of the big picture. As a result, many questions remain. Here we propose that Varroa's spread in Australia can be used to address them.

The non-native Australian honeybee population provides a final opportunity to collect pre-Varroa data on a large scale to understand the mechanisms and consequences of this spread [9]. As such, these data can be used to address a wide range of questions, ranging from those focused on better understanding Varroa's impacts on bees, to using Varroa as a model to understand fundamental processes in ecology and evolution. We briefly review previous findings and knowledge gaps highlighting opportunities in several distinct fields: evolutionary biology, virology and ecology, focusing on the function of both native and agricultural ecosystems.

## 2. Evolution

### (a) Coevolutionary dynamics in real time

Parasitic invasions provide opportunities to observe and quantify the role that genes play in driving evolution, relative to non-genetic extended phenotypes and extrinsic environmental cues—factors that continue to be hotly debated [7]. Host–parasite coevolutionary dynamics exert strong reciprocal selective pressures and provide insight into (i) how adaptive evolution occurs at varying timescales, depending on factors that affect mutation rates (e.g. organismal generation time, effective population size and genetic diversity) and (ii) whether phenotypic adaptation is the result of allele frequency changes at single or multiple loci. Population geneticists posit that a host's resistance response to parasitic pressure is akin to an ‘arms race’ deriving from respective selective sweeps at single loci influencing the trait of interest; whereas quantitative geneticists would argue that adaptation derives from minor allele frequency shifts across many loci—polygenic adaptation [10,11]. Yet, empirical evidence for either scenario is equivocal [11], and the recent incursion of Varroa into naive honeybee populations in Australia presents a valuable opportunity to track and understand how multi-faceted coevolved traits will respond to these selective pressures in a novel context.

Natural and selectively bred *A. mellifera* resistance has been demonstrated, but clear and consistent links between purported resistance traits and colony survival remain elusive [10,11]. Both selective sweeps on a single chromosome, as well as multiple gene/chromosome responses (identified via quantitative trait loci mapping or high-throughput sequencing of SNPs), have been inferred as genetic pathways to Varroa resistance, and while there are often mismatches between genetic markers and downstream function across research studies, commonalities in the neurology of olfactory pathways and behavioural traits do exist (see review Mondet *et al.* [12] and references therein). The Australian context presents multiple options to track host resistance by characterizing baseline levels of genetic diversity and directly measuring resistance as it occurs in real time among unmanaged colonies European honeybees *cf.* government-mandated control measures applied by apiarists; in parallel to the genomic profiles of the parasitic mites themselves [13].

### (b) Evolution of miticide resistances

**Figure 1.**
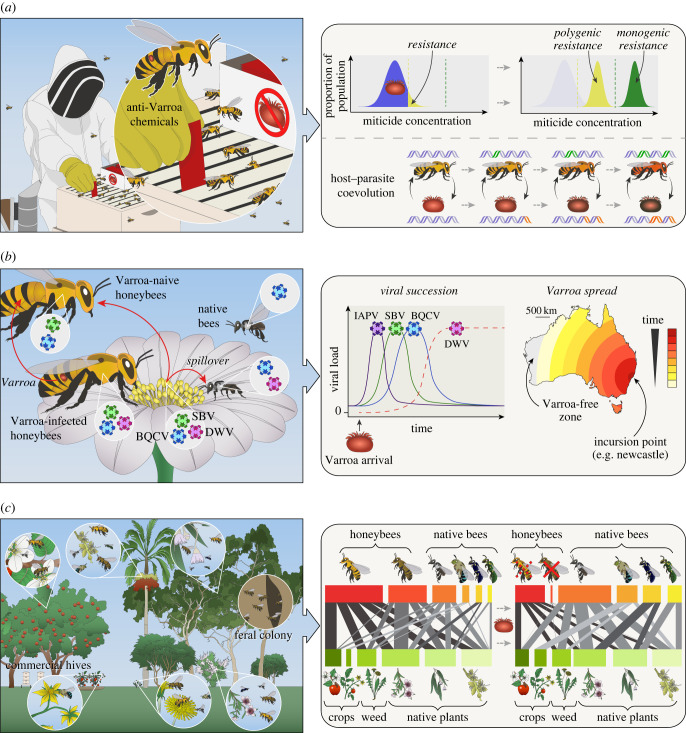
The three major expected impacts of Varroa on Australia's ecosystems. (*a*) Evolution. The recent incursion of *Varroa* into Australian naive honeybee populations enables evolutionary processes to be observed in both honeybees and Varroa. The use of anti-Varroa chemicals in hives, such as strips containing miticides, exposes Varroa to a selective pressure to adapt via miticide resistance mechanisms. The evolution of miticide resistance can contribute to our general understanding of adaptation, for the strength of selection (miticide concentration) will lead to different outcomes. For example, if the miticide concentration is not lethal to 100% of the original population, which has a normal distribution of viability (blue), the subsequent population will be formed from resistant individuals in the original population (yellow), with selection acting via the resistance phenotype on polygenic variation. At higher levels of miticide (dashed line), outside of the normal distribution of the original population. Selection will effectively act on rare mutations at single genes with a major impact on survival, over time leading to monogenic resistance (green) [14]. Similarly, bees and mites experience strong coevolutionary dynamics, providing general insights into this process. Monogenic and polygenic responses may occur in honeybees which facilitate natural adaptations to Varroa parasitism, or via artificial selective breeding programmes. This causes reciprocal genetic changes in the parasite and host over time. (*b*) Virology. Viral landscapes in bees change in the presence of Varroa. Mites facilitate a change in viral transmission route, leading to increased viral load and prevalence in honeybees. Viruses can spillover and impact viral landscapes in native bees that coexist in the same environments. As Varroa establishes and spreads, viral succession occurs with highly virulent viruses such as Black queen cell virus (BQCV) and Sacbrood virus (SBV) and Israeli acute paralysis virus/Kashmir bee virus (not shown) rapidly increasing, followed by Deformed wing virus (DWV) [8]. DWV is not yet established in Australia, which may result in unique outcomes if Varroa establishes in its absence. Tracking virus dynamics at the Varroa invasion front and over time in the colonized region, and observing viral load and disease emergence in honeybees and native bees will allow us to tease apart the relative influence of Varroa and viruses on bee health. (*c*) Ecology. The impact of Varroa on native and commercial ecosystems is largely driven by the removal of unmanaged honeybees, resulting in reduced pollination services. Pollination networks before and after Varroa establishment will change. Pollinators (left to right: commercial honeybee (*Apis mellifera*); unmanaged honeybee; native bees (*Tetragonula* sp.; *Amegilla* sp*.*; *Hylaeus* sp*.*; *Exoneura* sp*.*)) prior to Varroa are dominated by honeybees, but the near-complete removal of unmanaged bees after Varroa establishes will increase reliance on native bee pollination of crops and native plants (left to right: apple; tomato; dandelion; *Leptospermum*; *Eucalyptus*; native palm).

The evolution of pesticide resistance exemplifies rapid evolution in response to environmental change [14]. Most countries have implemented miticides to enable the continuation of the beekeeping industry [3]. The strength of selection (miticide concentration) might result in the evolution of either polygenic or monogenic mutations (figure 1). Collecting fine-scale geographical data on the quantitative use of miticides could therefore enable research on the mode of action and evolution of resistance. This is particularly true if the introduced Varroa strain lack miticide resistance.

First, detecting genetic changes due to miticide treatments might help us to understand the modes of action and to target new molecules or molecule groups for the development of new treatments. The modes of action of many pesticides are not well understood, and miticides are no exception (e.g. [15]). Second, the evolution of resistance to multiple pesticides is a complex issue for which little empirical data are available. Detecting the evolution of resistance to miticides that an introduced mite has not previously been exposed to could help us separate miticide classes by the functional mode of action and understand how pests can evolve resistance to multiple pesticides simultaneously [16].

The evolution of resistance by Varroa has been observed for most of the miticides leaving beekeepers with limited options to control the mite [17]. Varroa introductions to Australia are likely to be single-point entries, potentially even a single female, creating strong genetic bottlenecks. This should allow a complete characterisation of the miticide resistance spectrum at the point of introduction and the subsequent evolution of resistance. Introduced mites may have limited pre-existing resistance to miticides, making Australia an ideal system to study the evolution of chemical resistance.

## 3. Virology

### (a) Changes in viral community composition in the face of Varroa arrival: a model for viral competition and dynamics

The host range of RNA viruses is often limited by transmission opportunities [18]. In the absence of Varroa, viruses of honeybees predominantly occur at low levels, persisting in colonies as covert infections [19,20]. Virus transmission occurs mainly via the faecal–oral route through contact with contaminated food or infected individuals. The introduction of Varroa into a naive honeybee population creates a new transmission route, where viruses are injected directly through the cuticle as Varroa feeds, infecting sensitive tissues and life stages and circumventing the defences of the gut [21,22]. Viruses may also replicate in mites [13,23]. Mechanical and biological vectoring by Varroa drastically alters the honeybee viral landscape, leading to increased viral load and prevalence [13]. As Varroa spread throughout New Zealand, dynamic shifts in the prevalence of highly pathogenic viruses were observed at the Varroa invasion front, followed by the eventual establishment of Deformed wing virus (DWV) as the dominant virus [8] (figure 1). DWV was not detected in New Zealand before the arrival of Varroa, but has now reached near-ubiquity [24]. This matches observations elsewhere that if DWV is present, it will prevail as the dominant virus in heavily Varroa-infested colonies [7,25].

Australia is currently free from DWV [26], and there is no evidence from extensive molecular testing that it was introduced with the current Varroa incursion. Therefore, viral dynamics could differ from those observed elsewhere [26]. However, Australian honeybees possess a diverse virome with several Picornaviruses that are related to DWV [27], which could fill an evolutionary niche if they form associations with Varroa. Australia could still experience future DWV-carrying mite or bee incursions or introduce DWV by importing honeybee genetic material. Other invasive insects like ants and wasps can be reservoirs for viruses [28], providing additional entry routes for DWV into Australia, all of which would exacerbate the impacts of Varroa establishment. Comprehensive sampling of honeybees and native pollinators at the invasion front and throughout the colonized area, coupled with extensive pre-Varroa viral data [26,27] will enable the measurement of viral fluctuations if/when Varroa establishes and spreads (figure 1). Potential differences in viral landscapes between Australia and the rest of the world, particularly the absence of DWV, may then make it possible to tease apart the relative roles of viruses and mites in honeybee health and to identify whether interventions are required to combat viruses, rather than mites.

### (b) Viral evolution and host species range

Many viruses associated with honeybees, such as DWV, are generalist pathogens, able to colonize more than one host [29,30]. However, an important distinction lies between a genuine multi-host pathogen and a spillover event [31]. Pathogen spillover involves the transmission of a pathogen from a reservoir host to a new recipient species [32]. These events can be stochastic or transient if the pathogen cannot sustain transmission between individuals of the new host (spillover into ‘dead-end’ hosts; [33]). They may also represent the emergence of a novel pathogen in a different species [34–36]. Increased densities of commercial pollinators like honeybees and managed bumblebees, combined with the exacerbating effect of Varroa on virus levels, are thought to drive such spillover events in wild pollinators and contribute to population declines [37–39].

Viruses of honeybees have been detected in other bee species (and other arthropods) in several countries [40,41]. Even in Australia, common viruses of honeybees have been detected in native bees [42,43]. While there is evidence that some of these viruses can replicate in a range of bee species across several families [44–46], very few studies have investigated their pathogenic effects [47]. The high prevalence of DWV in honeybee populations has also made it the focus of most investigations of spillover to other insects. Studies of viral spillover for novel virus–host relationships provided by Australia's DWV-naive entomofauna, pre- and post-Varroa, can extend our understanding of how viruses of honeybees can impact wild insect communities.

## 4. Ecology

### (a) Interactions between additional stressors (Varroa and viruses) and agrochemicals

Global insect declines have been described as ‘death by a thousand cuts' because so many environmental stressors have been identified [48]. There is an ongoing controversy in the relative importance of the role played by stressors and in particular Varroa (and its associated viruses and miticide treatments) and agricultural pesticides in the decline of European honeybee health [49]. Elucidating these factors requires a Varroa-free control group, which is not possible in countries where it is long established. Cross-country experiments have allowed valuable comparisons between regions where Varroa is either present or absent, but are limited by strong confounding factors emanating from substantial environmental variation between countries [50].

As long as Varroa is not established in Australia, data on the effects of miticides and pesticides on bees in the absence of the mite can be collected and help inform local and worldwide public policies. The collection of adequate data on the survival and productivity of bee colonies before a potential Varroa establishment could allow comparisons with the susceptibility of bees to chemical stressors after a possible Varroa invasion.

If Varroa does establish in Australia, the progression of its spread might be sufficiently slow and patchy, as is typical for human-dispersed species, to observe the relative colony performance of Varroa-infested and Varroa-free hives. Testing a combination of control, treatment and interaction groups [50] (by exposing hives to a relevant mixture [51] of miticides, pesticides and Varroa) is crucial to determine their relative effects on bee colonies. Direct evidence of the relative roles of environmental stressors will be crucial to prioritize areas of research likely to deliver the greatest benefits for bee health for decades to come.

### (b) Impact of the removal of introduced pollinators on native ecosystems

The lack of data on pollinator communities before Varroa incursions in other countries means that we have little idea of how Varroa and subsequent unmanaged honeybee declines have impacted pollinator community structure, species abundance and plant reproduction, or the transience of these impacts [9]. A reduction in unmanaged honeybee populations as a result of Varroa [52] is likely to have a range of complex and cascading effects on native ecosystems (figure 1). Introduced honeybees are abundant flower visitors in many Australian landscapes, particularly in the temperate southern parts of the continent [53,54]. High densities of honeybees can suppress or displace native bees via competition for floral resources [55,56], and such competitive exclusion has been demonstrated in other parts of the world [57,58].

Changes in honeybee abundance could have important consequences for plant communities in natural ecosystems, either directly (via pollination from honeybees themselves) or indirectly (via changes in the behaviour or prevalence of other pollinators) [59]. Therefore, a key question is whether Varroa-mediated change in unmanaged honeybee populations will occur, and if so, whether it will correspond to qualitative and quantitative changes in the community of native pollinators or native plants. Baseline data are missing for most of Australia's vegetation types and so monitoring bee and plant communities in different ecosystems pre- and post-unmanaged honeybee decline is imperative to quantify these effects (e.g. [60,61]). Such data could be collected through formalized and standardized sampling of plant–pollinator networks across different ecosystems on an annual basis, tracking changes in measures like niche overlap and reproductive fitness (e.g. [62])

In addition, unmanaged honeybees compete with certain birds and mammals for nesting hollows [63], and, during times of low supply, possibly also for nectar [55,64]. Documenting changes in bird and unmanaged honeybee populations simultaneously would allow us to quantify the impact of unmanaged honeybees on these vertebrates.

### (c) Impact of the loss of introduced pollinators on crop productivity

Australia has a large unmanaged honeybee population (e.g. [62]). The contribution of this population to the pollination of crops in Australia is largely unquantified [62]. The loss of unmanaged honeybee colonies from Australian landscapes (e.g. [49,65,66]; figure 1) may result in affected industries having to pay for pollination services or face reductions in the quality and quantity of yield [67]. Particularly at risk in Australia are apples, berries and macadamia crops [68–70]. For key crops in tropical and subtropical regions, social stingless bees (Meliponini) have the potential to meet future pollination needs [71,72], but significant research investment is still needed to develop these native bees commercially [73]. Quantification of bee populations and production across the invasion front over time will elucidate the commercial benefit of unmanaged honeybees in Australia.

Pollination services have largely been taken for granted in Australia, with little development of management techniques to improve honeybee pollination depending on the crop [74,75]. A potential reduction in honeybee colonies available for pollination services should drive investment in research to improve the efficiency and effectiveness of these services [74,76]. At the same time, experiments with non-*Apis* pollinators in Australia can pave the way for diversifying the pool of insects providing agricultural pollination services worldwide and a lower reliance on an introduced species.

## 5. Summary

Australia has long been an unwitting laboratory for ecological experimentation. Some of these experiments, like the failed attempt to use cane toads to control cane beetles, have become infamous. Others, such as the control of invasive rabbits using the Myxoma virus, are now textbook examples of evolutionary biology in action. The arrival of Varroa mites in Australia has been forecasted to carry a heavy cost for industry, threatening the livelihoods of many people. Yet, in the face of this calamity, we hope that the Varroa incursion can be seen as an opportunity to apply global knowledge about the impacts of Varroa to local Australian conditions, and to minimize the harm caused by yet another introduction of yet another invasive pest. At the same time, ecological and evolutionary information gained in Australia should be broadly informative for a range of biological disciplines worldwide. Tackling these large-scale questions requires an urgent need for collaboration both within Australia and internationally, deploying collaborative research initiatives before it is too late.

## Data Availability

This article has no additional data.
